# Treatment of concomitant persistent chylothorax and superior vena cava syndrome through innominate vein-right atrial bypass

**DOI:** 10.1093/icvts/ivae176

**Published:** 2024-10-28

**Authors:** Emrah Şişli, Arzu Funda Tarhan, Eylem Kıral, Gürkan Bozan

**Affiliations:** Section of Paediatric Cardiovascular Surgery, Department of Cardiovascular Surgery, Osmangazi University Faculty of Medicine, Eskişehir, Türkiye; Section of Paediatric Cardiovascular Surgery, Department of Cardiovascular Surgery, Osmangazi University Faculty of Medicine, Eskişehir, Türkiye; Section of Paediatric Intensive Care, Department of Paediatrics, Osmangazi University Faculty of Medicine, Eskişehir, Türkiye; Section of Paediatric Intensive Care, Department of Paediatrics, Osmangazi University Faculty of Medicine, Eskişehir, Türkiye

**Keywords:** Brachiocephalic veins, Thrombosis, Chylothorax, Superior vena cava syndrome, Heart bypass, right

## Abstract

Persistent chylothorax is a major challenge in paediatric patients. We present a case of a 6.5 kg, 1-year-old boy with superior vena cava syndrome and persistent chylothorax who underwent successful surgery without cardiopulmonary bypass. His medical history included multiple comorbidities such as myeloproliferative disease, short bowel syndrome and central vein catheterizations. The patient also had innominate vein thrombosis, progressing to superior vena cava, and was on anticoagulants. Despite dietary changes and somatostatin, his high-output chylous pleural effusion persisted. He was treated with innominate vein-to-right atrial bypass using a 6-mm Dacron graft. Postoperatively, there was a significant reduction in effusion and accelerated recovery. Somatostatin failure was likely due to mechanical obstruction of the thoracic duct.

## INTRODUCTION

Persistent chylothorax, often iatrogenic following congenital cardiac surgery, is a major challenge in paediatric patients. We present a case with multiple comorbidities, persistent chylothorax and superior vena cava syndrome (SVCS), successfully treated with left brachiocephalic vein-to-right atrial bypass without cardiopulmonary bypass (CPB).

## CASE REPORT

**Figure 1:**
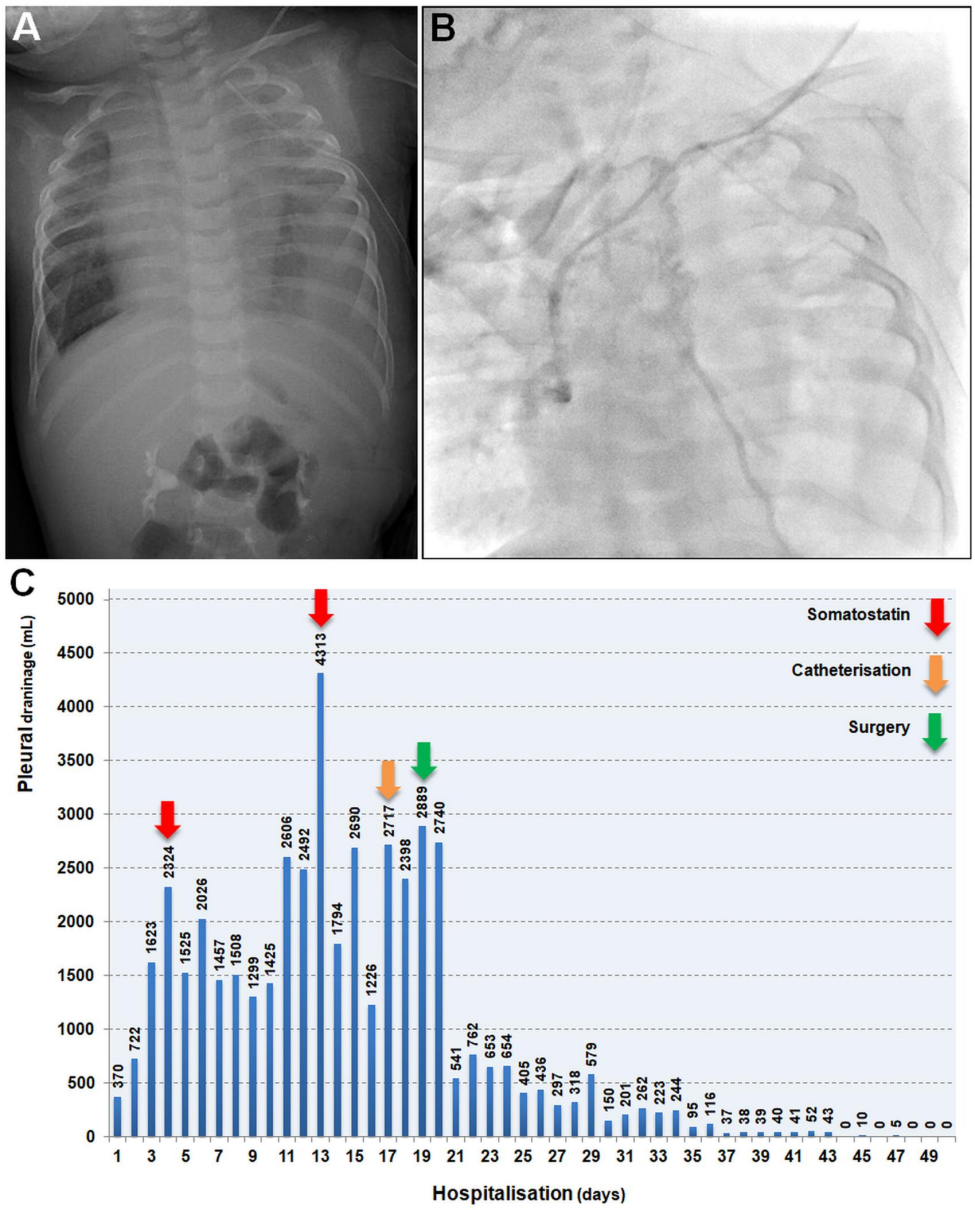
Preoperative chest X-ray revealing left massive chylothorax (**A**) and catheterization showing occluded left Pirogoff angle and innominate vein (**B**). (**C**) The amount of chylous pleural drainage along with initiation points of treatment modalities on the last admission.

A 6.5 kg, 1-year-old boy with SVCS presented with persistent left chylothorax and increasing head and upper extremity oedema over the past month. His history included multiple hospital admissions, myeloproliferative disease, short bowel syndrome and multiple central vein catheterizations for long-term intravenous medications and total parenteral nutrition. He had been on anticoagulation for innominate vein (IV) thrombosis, which progressed to the superior vena cava (SVC), secondary to multiple permanent port implantations. Despite medical treatment with somatostatin and autologous blood pleurodesis, his chylothorax persisted, requiring multiple tube thoracostomies. Chest X-ray showed massive left pleural effusion, lung collapse and mediastinal shift (Fig. 1A). Catheterization via the left subclavian vein revealed high venous pressure (29 mmHg) and an occlusion at the junction of the left jugular and subclavian veins, extending to the IV and SVC (Fig. 1B). Despite somatostatin treatment, chylous drainage peaked at over 4 l per day (Fig. 1C). The deficit was aggressively replaced with dietary modifications, oral rehydration solutions, intravenous fluids and fat-free total parenteral nutrition by paediatric intensivists.

**Figure 2:**
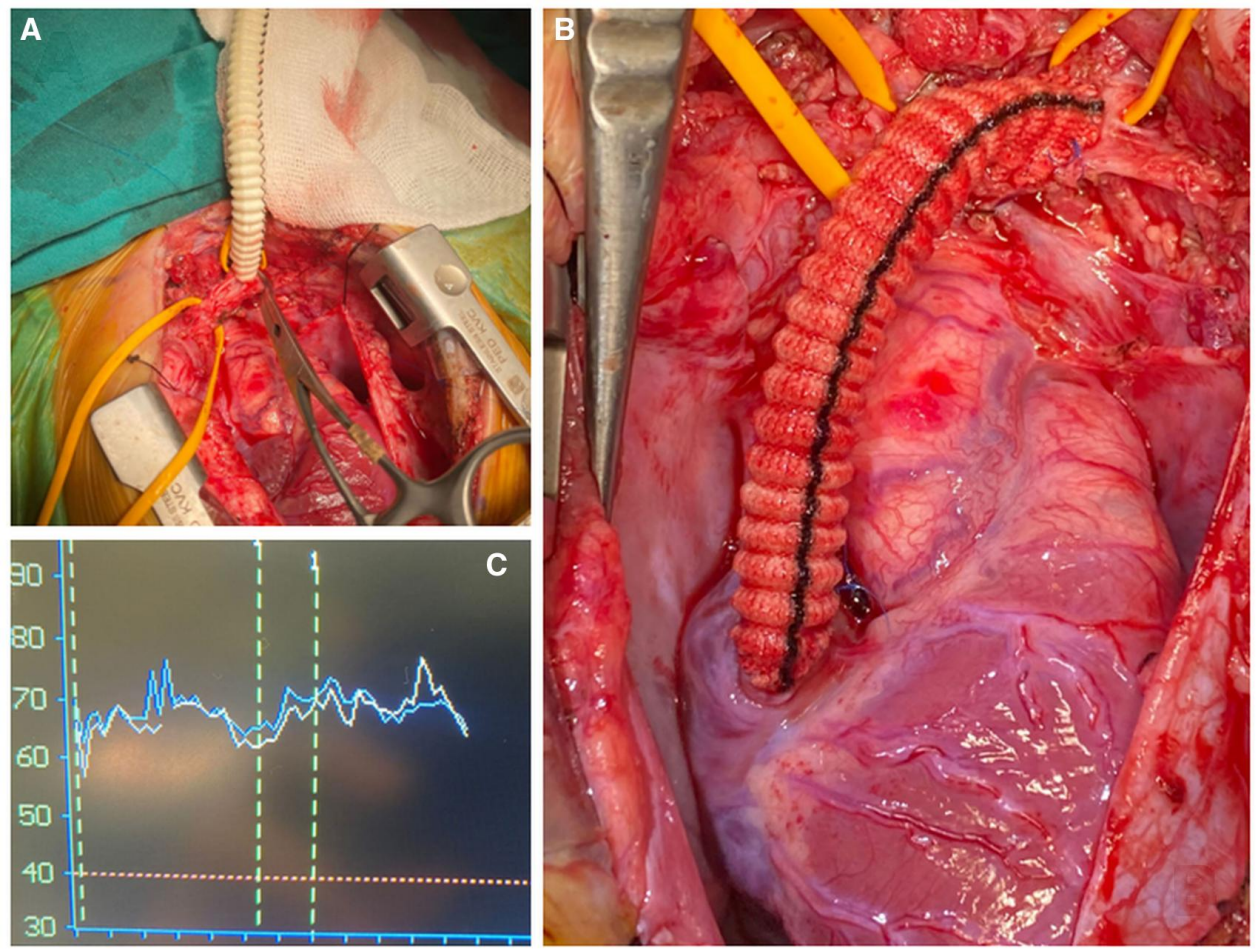
Intraoperative view revealing clamping of the left Pirogoff angle (**A**) and completion of the innominate vein to right atrial bypass using 6 mm Dacron tube graft (**B**). (**C**) Perioperative near-infrared spectroscopy monitoring.

During surgery, following median sternotomy and thymectomy, the IV and SVC junction, which contained a well-organized thrombus, was explored. Innominate vein exploration was extended to the left Pirogoff angle. Considering the multiple comorbidities, CPB was avoided, although kept on standby. After systemic heparinization, a side-biting clamp was applied, and the thrombus was removed with difficulty. A 6-mm Dacron graft was anastomosed to the Pirogoff angle, with the distal anastomosis to the right atrial appendix (Fig. 2A and B). Cerebral perfusion was monitored throughout, with no significant drops observed (Fig. 2C).

Postoperatively, the patient’s oedema and chylous drainage decreased significantly, leading to an uneventful recovery. The patient was discharged on acetylsalicylic acid and warfarin. One year later, the chylothorax had not recurred, the graft remained patent and no swelling was present.

## DISCUSSION

The management of persistent high-output chylothorax necessitates multidisciplinary approach with a variety of treatment steps including dietary modifications, intravenous somatostatin, chemical pleurodesis, and finally, either interventional or surgical procedures [1–3]. In our case, besides SVCS and persistent chylothorax, there were multiple considerable comorbidities.

Today, interventional procedures are not only widely applied but also recommended in the first-line before surgery with satisfactory outcomes [1, 3]. However, any interventional procedure was not opted in our case, due to the chronic nature of the SVC occlusion and the growth potential of the case. Additionally, although thoracic duct (TD) can effectively be occluded to prevent occurrence of chylothorax [3], preclusion of the flow of chyle comprises long-term consequences, such as chronic diarrhoea, which would be the last malady in our case because of the short bowel syndrome. Thus, TD ligation was reserved as a last resort.

Hraška *et al.* [4] introduced a novel surgical approach, the IV turn-down procedure, to relieve systemic venous hypertension that impairs lymphatic drainage via the TD. This technique involves dividing the IV and connecting it to the left or right atrial appendix, alleviating persistent chylous pleural effusions refractory to treatment, as in our case. It was apparent that the failure of somatostatin in our patient was due to mechanical occlusion of the TD’s runoff.

As a tip, since there are collateral pathways from the supra-cardiac veins, venous return can be safely occluded with a clamp if cerebral saturation is monitored and CPB backup is available. Most SVC reconstructions today are performed without CPB [5], minimizing the inflammatory burden. Additionally, in chronic cases, a surgeon may avoid thrombectomy or atherectomy due to the high risk of injury from an organized thrombus adhering to the fragile vessel wall.

While a PTFE graft is recommended for venous placement, non-ringed versions are more prone to kinking in the mediastinum. Dacron grafts, which stiffen when soaked, may offer a superior alternative. In our view, using foreign materials instead of autologous pericardium [5] is less favourable due to thrombosis risk. However, the procedure discussed here presents a potential new treatment option for selected patients with multiple comorbidities.

In conclusion, the procedure can safely be applied with ease in select cases in whom SVCS is associated with refractory persistent chylothorax. Besides having the potential to be a treatment option in these cases, the procedure seems to be beneficial in terms of remediating persistent chylothorax and SVCS in cases with multiple comorbidities.

## Data Availability

The data underlying this article are available in the article.

## Notes

Presented (SP-03) at the 22^nd^ National Congress of Paediatric Cardiology and Cardiac Surgery, which was held in Antalya, Türkiye on 7–10 March 2014.
